# Platelet-rich plasma hydrogel supports long-term germ cell maintenance in rat testicular organotypic culture

**DOI:** 10.1007/s10815-026-03936-9

**Published:** 2026-06-24

**Authors:** Nilgün Öksel, Merve Bulut Özer, İlayda Yücel, Muzaffer Katar

**Affiliations:** 1 Faculty of Medicine, Department of Histology and Embryology, Tokat Gaziosmanpaşa University, Tokat Province, Turkey; 2 Faculty of Medicine, Department of Medical Biochemistry, Tokat Gaziosmanpaşa University, Tokat Province, Turkey

**Keywords:** Rat testicular organotypic culture, In vitro spermatogenesis PRP hydrogel

## Abstract

**Purpose:**

Fertility preservation has become an important clinical concern for prepubertal boys undergoing gonadotoxic cancer treatments. Preservation of germ cells within immature testicular tissue is therefore considered a promising strategy for future fertility restoration. The purpose of this study was to evaluate the effects of platelet-rich plasma (PRP), used as a culture medium supplement and/or as a hydrogel scaffold, on germ cell survival and spermatogenic progression in a three-dimensional rat testicular organotypic culture system and to explore its potential relevance for fertility preservation approaches.

**Methods:**

Testicular tissue fragments obtained from postnatal day 7–9 Wistar albino rats were cultured using an air–liquid interface organotypic culture system on agarose blocks. PRP was incorporated either as a hydrogel scaffold supporting the tissue fragments or as a soluble supplement in the culture medium. Cultures were maintained for 8 weeks, and samples were collected at weeks 1, 3, 4, 6, and 8. Spermatogenic progression was evaluated using histomorphometric and immunohistochemical analyses.

**Results:**

SCP3( +) spermatocytes were better preserved in PRP hydrogel–supported cultures, with persistence observed up to week 8 only in this group. In addition, PLZF( +) undifferentiated spermatogonia remained detectable at week 8 in PRP hydrogel cultures, indicating improved preservation of the spermatogonial population. In vitro spermatogenesis progressed up to the spermatocyte stage in all experimental conditions, whereas differentiation into round spermatids was not observed.

**Conclusions:**

This organotypic culture approach may support germ cell maintenance during long-term culture and may contribute to the development of future fertility preservation strategies.

## Introduction

Spermatogenesis is a highly regulated process involving the differentiation of spermatogonia into mature spermatozoa. Gonadotoxic cancer treatments, including chemotherapy and radiotherapy, can severely disrupt this process and often lead to permanent infertility. This issue is particularly important for prepubertal boys, as the germ cell population in their testes is restricted to immature stages; sperm cryopreservation cannot be performed before treatment [1, 2]. An estimated 200.000 children under the age of 15 are diagnosed with cancer globally each year, and advances in treatment have increased survival rates to nearly 80%. Accordingly, infertility has become a major long-term concern affecting the quality of life of cancer survivors [3]. For this reason, the development of effective fertility preservation strategies for patients exposed to gonadotoxic therapies has become an important focus in reproductive medicine. Among the experimental approaches proposed for fertility preservation in prepubertal patients, the in vitro maturation of immature testicular tissue using testicular organotypic culture systems represents one of the most promising strategies. These culture systems aim to recreate key aspects of the testicular microenvironment in vitro, enabling germ cells within immature testicular fragments to progress through spermatogenesis outside the body. By preserving cell–cell interactions and maintaining the structural organization of seminiferous tubules, organotypic culture platforms provide valuable models for studying spermatogenic progression and for evaluating factors that may support germ cell survival and differentiation [1].

The most successful demonstrations of complete in vitro spermatogenesis have been reported in mouse models. However, translating these findings to other species has proven considerably more challenging. In particular, studies investigating rat testicular organotypic culture systems remain limited [4–10], and most have reported that spermatogenic progression can be maintained only up to the spermatocyte stage despite various modifications of culture conditions. Given that the rat is one of the most widely used experimental models in biomedical research, the establishment and optimization of reliable rat-based organotypic culture platforms are of considerable importance. Such models may provide valuable insights into species-specific aspects of spermatogenesis and may contribute to the development of clinically relevant fertility preservation strategies [7].

Platelet-rich plasma (PRP) has recently gained considerable attention in regenerative medicine due to its high concentration of growth factors and cytokines that support cell survival, proliferation, and tissue repair. As a biologically active autologous product, PRP is enriched with essential mediators released from platelet α-granules upon activation, including fibroblast growth factor (FGF), vascular endothelial growth factor (VEGF), transforming growth factor-β1 (TGF-β1), and platelet-derived growth factor (PDGF). These mediators play fundamental roles in angiogenesis and extracellular matrix production, along with cellular migration and differentiation. Importantly, PRP represents a xeno-free biological product, making it particularly attractive for translational and clinical applications in which the avoidance of xenogenic components is critical [11, 12].

Accumulating evidence from experimental models demonstrates that PRP exerts regenerative effects on testicular architecture and spermatogenesis following tissue injury [13–21]. Furthermore,in vitro observations have shown that PRP supplementation enhances spermatogonial stem cell (SSC) proliferation, promotes colony formation, and preserves key stem cell characteristics [22, 23]. Only a few studies have also explored the use of PRP as a soluble culture medium supplement in testicular organotypic culture systems, primarily in mouse models using human-derived PRP [24, 25]. However, the structural potential of PRP within such systems has remained largely underexplored.

Beyond its role as a soluble additive, activated PRP can form a fibrin-based hydrogel scaffold characterized by a three-dimensional (3D) network structure and high water content. This matrix facilitates nutrient diffusion while providing mechanical support capable of recreating aspects of the complex testicular microenvironment required for germ cell maintenance and spermatogenic progression. Despite these promising properties, the potential role of PRP-derived hydrogel scaffolds in supporting testicular tissue architecture and germ cell survival in rat organotypic culture systems has not yet been thoroughly investigated. Therefore, the present study aimed to evaluate whether a PRP-based 3D microenvironment could support germ cell maintenance and spermatogenic progression in a rat testicular organotypic culture model.

In the present study, we evaluated the effects of species-matched rat PRP, utilized both as a culture medium supplement and as a hydrogel scaffold, within a rat testicular organotypic culture system. By examining germ cell markers and seminiferous tubule morphology during long-term culture, we investigated the effects of PRP-based culture conditions on germ cell maintenance and spermatogenic progression in a three-dimensional rat testicular organotypic culture model. Understanding the role of PRP in supporting in vitro spermatogenesis may contribute to the development of xeno-free and clinically translatable culture platforms for fertility preservation and reproductive medicine, particularly for prepubertal cancer patients who have no established fertility preservation options.

## Materials and method

### Experimental design

**Fig. 1:**
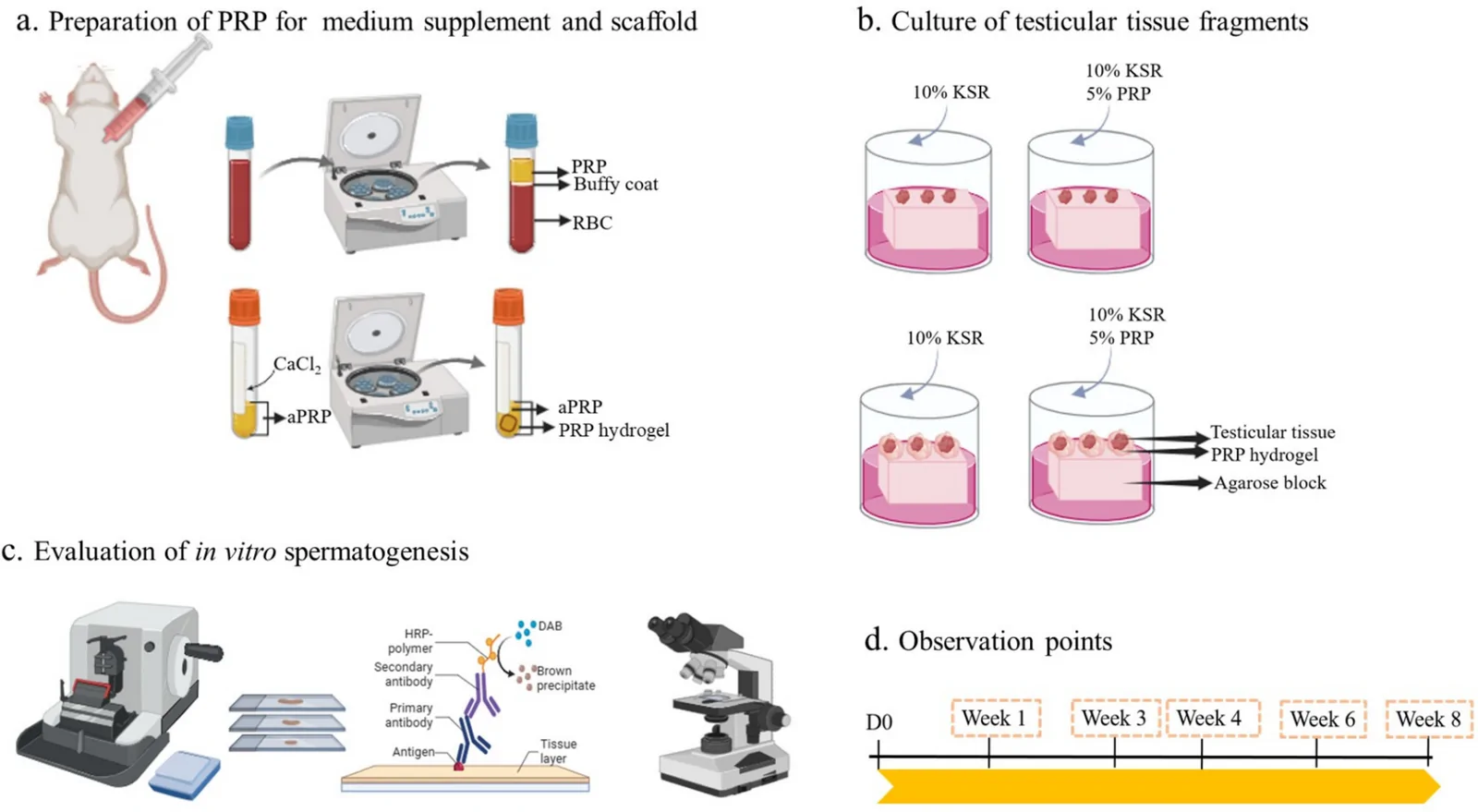
Schematic representation of the experimental design. (**a**) Preparation of platelet-rich plasma (PRP) for culture medium supplementation and PRP hydrogel scaffold production. (**b**) Culture of rat testicular tissue fragments in agarose-based air–liquid interface organotypic culture systems with PRP supplementation and/or PRP hydrogel scaffolds. (**c**) Evaluation of cultured testicular tissues by histomorphometric and immunohistochemical analyses. (**d**) Observation and sampling time points during the 8-week culture period

The overall experimental workflow of the study is illustrated in Fig. 1. This experimental study was designed to evaluate the effects of rat-derived PRP, used either as a culture medium supplement or as a hydrogel scaffold, on germ cell survival and spermatogenic progression in a rat testicular organotypic culture system. The experimental design included four experimental groups with six biological replicates per group. Testicular tissue fragments obtained from postnatal day 7–9 Wistar albino rats were cultured using an air–liquid interface organotypic culture platform for up to 8 weeks. Samples were collected at five observation time points (weeks 1, 3, 4, 6, and 8) for histological, histomorphometric, and immunohistochemical analyses. The use of animal material and all experimental procedures were approved by the Tokat Gaziosmanpaşa University Animal Experiments Local Ethics Committee (Approval No.: 2024-HADYEK-09).

### Culture method

Wistar albino rats, aged prepubertal P7–P9, were euthanized and immature testes were removed. After removal, the testes were decapsulated and mechanically divided into approximately 6–8 fragments per testis using forceps. Tissue fragments obtained from each donor animal were randomly distributed among all experimental groups to minimize potential developmental bias. The fragments were set onto 1.5% agarose gel supports within 12-well plates containing 0.65 mL of culture medium in each well. To prepare the culture stands, agarose was dissolved in distilled water (1.5% wt/vol) and autoclaved. The 30 mL of agarose solution was poured into 9-cm dishes to obtain gels approximately 5 mm thickness, then cut into about 10 × 10 × 5 mm pieces, which served as support platforms for the tissue fragments. These blocks were kept in culture media for 24 h to replace the water content prior to the experiment. These blocks were kept in culture medium for 24 h prior to the experiment to allow replacement of water in the agarose with the culture medium. Each gel stand was loaded with three tissue fragments. The culture medium was replaced every other day, and the testicular fragments were cultured at 34 °C in a humidified 5% CO_2_ atmosphere.

### Culture medium composition and experimental groups

All experimental groups were maintained in the same basal culture medium. The culture medium was composed of α-MEM (MEM-RXA, Capricorn, Germany) supplemented with 10% KnockOut Serum Replacement (10,828,028, Thermo Fisher Scientific, USA), 1% antibiotic–antimycotic (AAS-B, Capricorn, Germany), 0.1% gentamicin, 1% ITS-G (Capricorn, Germany), and 1% L-glutamine. In addition, the following components were included to reach the indicated final concentrations: 1 μM retinoic acid (R2625, Sigma, USA, 25 mM stock solution in DMSO), 1 μM retinol (R7632, Sigma, USA, 25 mM stock solution in DMSO), 0.1 μM L-ascorbic acid (A5960, Sigma, USA, 10 mM stock solution in DDW), 1.0 ng/mL FSH (F4021, Sigma, USA, 0.6 μg/mL stock solution in DDW), 1.0 ng/mL LH (L6420, Sigma, USA, 3.33 μg/mL stock solution in DPBS + 1 mg/mL BSA), 23.2 μM α-tocopherol (T3251, Sigma, USA, 1 M stock solution in DMSO), 1.0 μM testosterone (GLP-GC45935, 50 mM stock solution in DMSO), and 2 ng/mL triiodothyronine (T6397, Sigma, USA, 6.6 μg/mL stock sol. in DMSO).

Using this basal medium, four experimental groups were established to evaluate the role of rat-derived PRP as either a media supplement, a hydrogel scaffold, or a combination of both:

- **Group 1 (control):** Testicular fragments cultured on agarose blocks in standard culture medium.
- **Group 2 (PRP):** Testicular fragments cultured on agarose blocks in culture medium supplemented with 5% activated PRP.
- **Group 3 (PRP hydrogel):** Testicular fragments cultured on PRP hydrogel scaffolds in standard culture medium.
- **Group 4 (PRP-PRP hydrogel):** Testicular fragments cultured on PRP hydrogel scaffolds in culture medium supplemented with 5% activated PRP.

### Preparation of activated PRP and PRP gel

Cardiac blood was collected aseptically from 8- to 10-week-old male Wistar albino rats. Approximately 6–8 mL of blood per animal was transferred into tubes containing anticoagulant (3.2% sodium citrate), and centrifuged at 830 × g for 10 min. After centrifugation, the platelet-rich fraction containing the buffy coat was placed in a tube without an anticoagulant. Subsequently, PRP was incubated with 10% CaCl_2_ (50 µL/mL) at 37 °C for 10 min, followed by 20 min at room temperature to activate the platelets. After platelet degranulation, the PRP gel was collected and the supernatant containing growth factors was collected. The collected PRP gels were cut into pieces measuring 2–3 mm, and a single tissue fragment was placed on each piece. Three PRP gel pieces were placed onto agar blocks and cultured using the air–liquid interface technique. The supernatant was used as a culture medium at a 5% concentration.

### Histological and immunohistochemical examinations

Organotypic cultures were maintained for 8 weeks, and samples were collected at weeks 1, 3, 4, 6, and 8 for histological evaluation. Cultured testicular fragments were fixed in Bouin’s solution, embedded in paraffin, and sectioned. For histological assessment, paraffin sections were processed for hematoxylin–eosin (H&E) and periodic acid–Schiff (PAS) staining. Germ cell developmental stages were identified based on nuclear morphology and characteristic staining patterns. Additionally, seminiferous tubule area was quantitatively measured to evaluate structural preservation and spermatogenic progression.

Immunohistochemical analyses were performed to identify and localize germ cell populations at different stages of spermatogenesis using stage-specific markers. Bouin-fixed sections were deparaffinized, rehydrated, and treated with 0.5% lithium carbonate and 100 mM glycine. Antigen retrieval was performed at 95 °C for 90 min in Tris–EDTA buffer for SCP3 or citrate buffer (PL-1000-CTB, PatoLab, Turkey) for PLZF and ACR. Endogenous peroxidase activity was suppressed by 0.3% hydrogen peroxide treatment for 30 min. This was followed by a blocking step with a mixture of 3% BSA, 5% normal goat serum, and 0.05% Triton X-100 in PBS to reduce non-specific binding. The sections were subjected to primary antibodies against promyelocytic leukemia zinc finger protein (PLZF), a marker of undifferentiated spermatogonia (1:100) (SC-28319, Santa-Cruz (synaptonemal complex protein 3), a meiotic germ cell marker involved in synaptonemal complex formation (1:250) (PA1-16,766, Invitrogen, USA); and ACR (Acrosin), an acrosomal protease expressed in late spermatids and spermatozoa (1:2500) (NBP2-14,260, Novus, USA). All primary antibodies were applied overnight at 4 °C. After washing, sections were treated with HRP-labeled secondary antibody (ab236466, Abcam, UK) for 45 min at room temperature. Visualization was performed using DAB chromogen (PL-060-HDX, PatoLab, Turkey) and counterstaining with Mayer’s hematoxylin for 20 s. Following dehydration through a graded ethanol series and xylene clearing, slides were mounted and visualized using a Nikon Eclipse E2000 light microscope equipped with a Nikon DS-Fi1 camera. Quantification of immunohistochemical staining was based on the percentage of seminiferous tubules exhibiting PLZF(+), SCP3(+), or ACR(+) germ cells out of the total tubules examined in each section. These values were then compared among different culture conditions.

### Statistical analysis

Statistical analyses were performed via one-way ANOVA followed by Bonferroni post hoc tests using SPSS v.22. Data are expressed as mean ± standard deviation (SD), with statistical significance defined at *p* < 0.05.

## Results

### PRP hydrogel promoted tubular growth and lumen-like structure formation in rat testicular organotypic culture

**Fig. 2:**
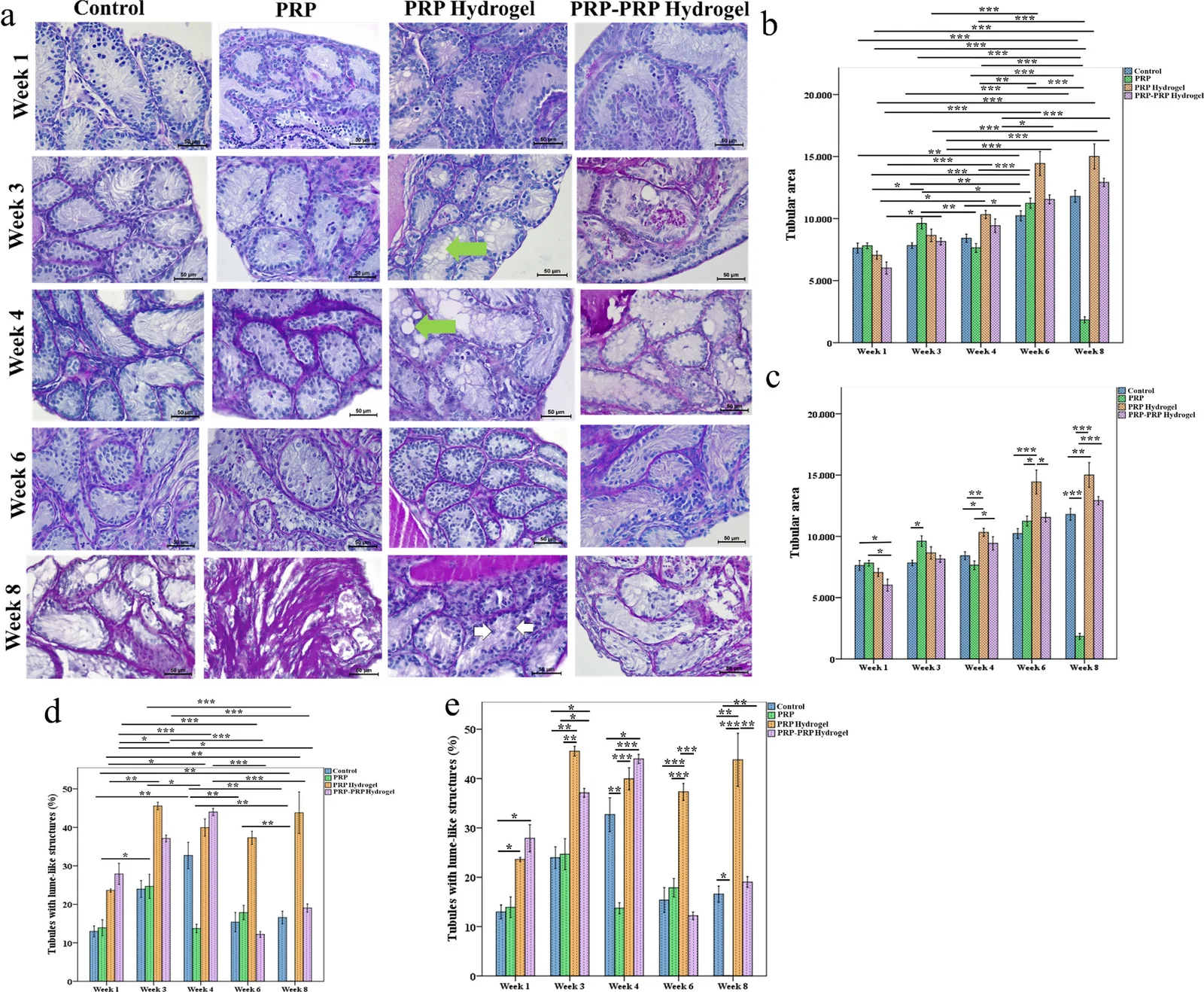
Morphometric analysis of tubular areas and histological evaluation. (**a**) Representative images of PAS-stained testicular sections for control, PRP, PRP hydrogel, and PRP-PRP hydrogel groups. Green arrows show lumen-like intratubular structures observed in PRP hydrogel cultures at weeks 3 and 4 and white arrows indicate the presence of spermatocytes in the PRP hydrogel group at week 8. Scale bar = 50 µm. (**b**) Quantitative evaluation of time-dependent alterations in tubular area within each experimental group. (**c**) Quantitative inter-group comparison of tubular areas between experimental groups at weeks. (**d**) Time-dependent changes in the percentage of seminiferous tubules containing lumen-like intratubular structures within each group.** e** Comparison of lumen-like structure-containing seminiferous tubules between experimental groups at weeks. Data are presented as mean ± SD (*n* = 6 per group). Statistical significance is indicated as *p < 0.05, **p < 0.01, and ***p < 0.001

Seminiferous tubular area showed time-dependent changes across experimental groups during the culture period. In the control group, tubular area remained relatively stable between weeks 1 and 4. A noticeable increase was observed at week 6 (*p* = 0.024), after which no further change was detected between weeks 6 and 8. In the PRP group, tubular area showed an increase between weeks 1 and 3 (*p* = 0.011), with a subsequent decline from week 3 to week 4 (*p* = 0.005). Although an increase was observed at week 6 (*p* < 0.001), the tubular area significantly decreased by week 8. In the PRP hydrogel group, tubular area remained relatively stable up to week 4, with values at week 4 being slightly higher than those at week 1 (*p* = 0.027). A marked increase was observed at week 6 (*p* = 0.003), and tubular area was sustained through week 8. In the PRP-PRP hydrogel group, tubular area increased from week 1 to week 3 (*p* = 0.012) and showed no notable change at week 4. A marked increase was observed at week 6 (*p* = 0.013), and tubular area remained stable until week 8 (Fig. 2a and b).

At week 1, tubular area did not differ significantly among the control, PRP, and PRP hydrogel groups, while the PRP-PRP hydrogel group demonstrated a decrease in tubular area relative to the control (*p* = 0.038) and PRP groups (*p* = 0.017). At week 3, the PRP group exhibited a higher tubular area compared with the control group (*p* = 0.020), while the PRP hydrogel and PRP-PRP hydrogel groups showed tubular areas comparable to those of the control and PRP groups. At week 4, the PRP hydrogel group exhibited a higher tubular area compared with the control (*p* = 0.018) and PRP (*p* = 0.001) groups, whereas no difference was observed between the PRP hydrogel and PRP-PRP hydrogel groups. At week 6, tubular area was highest in the PRP hydrogel group, with values higher than those of the control (*p* < 0.001), PRP (*p* = 0.007), and PRP-PRP hydrogel groups (*p* = 0.016), whereas no difference was observed among the control, PRP, and PRP-PRP hydrogel groups. By week 8, the PRP hydrogel group continued to exhibit a significantly higher tubular area compared with the control (*p* = 0.006) and PRP groups (*p* < 0.001), whereas the PRP group displayed the lowest tubular area among all experimental groups (Fig. 2a and c).

The percentage of seminiferous tubules containing lumen-like intratubular structures showed dynamic changes throughout the culture period in all experimental groups. In the control group, the percentage of lumen-like structure-containing tubules increased significantly from week 1 to week 4 (*p* = 0.001), followed by a significant decrease between weeks 4 and 6 (*p* = 0.004). No significant change was observed between weeks 6 and 8. In the PRP group, the percentage increased from week 1 to week 3 (*p* = 0.030) and subsequently decreased between weeks 3 and 4 (*p* = 0.027), with no significant differences detected between weeks 4 and 6. No lumen-like intratubular structures were observed at week 8 in the PRP group. In the PRP hydrogel group, the percentage of lumen-like structure-containing tubules increased significantly from week 1 to week 3 (*p* = 0.002), and no further significant changes were observed thereafter. Notably, the percentages observed at weeks 4 and 8 remained significantly higher compared with week 1 (*p* = 0.019 and *p* = 0.004, respectively). In the PRP-PRP hydrogel group, the percentage increased significantly from week 1 to week 3 (*p* = 0.013), remained relatively stable between weeks 3 and 4, and decreased at week 6 (*p* < 0.001). No significant difference was observed between weeks 6 and 8.

At week 1, the percentages of seminiferous tubules containing lumen-like intratubular structures were significantly higher in the PRP hydrogel and PRP-PRP hydrogel groups compared with the control and PRP groups (PRP hydrogel vs. control: *p* = 0.024; PRP hydrogel vs. PRP: *p* = 0.039; PRP-PRP hydrogel vs. control: *p* = 0.003; PRP-PRP hydrogel vs. PRP: *p* = 0.005). Comparable percentages were observed between the PRP hydrogel and PRP-PRP hydrogel groups. At week 3, findings similar to those observed at week 1 were detected. The percentages of seminiferous tubules containing lumen-like intratubular structures remained significantly higher in the PRP hydrogel group compared with the control (*p* = 0.001) and PRP (*p* = 0.001) groups. The PRP-PRP hydrogel group also showed significantly higher percentages than the control (*p* = 0.012) and PRP (*p* = 0.016) groups. No significant difference was detected between the two hydrogel-supported groups. During week 4, the percentage of lumen-like structure-containing tubules was significantly higher in the PRP hydrogel group compared with the PRP group (*p* < 0.001). The PRP-PRP hydrogel group showed significantly higher values than the control (*p* = 0.038) and PRP (*p* < 0.001) groups. At week 6, the PRP hydrogel group demonstrated significantly higher percentages than all other experimental groups (*p* < 0.001 for all comparisons). Similarly, at week 8, the PRP hydrogel group maintained significantly higher percentages compared with the control (*p* = 0.001), PRP (*p* < 0.001), and PRP-PRP hydrogel (*p* = 0.002) groups.

### *PRP hydrogel supported the long-term maintenance of PLZF(* +*) undifferentiated spermatogonia*

**Fig. 3:**
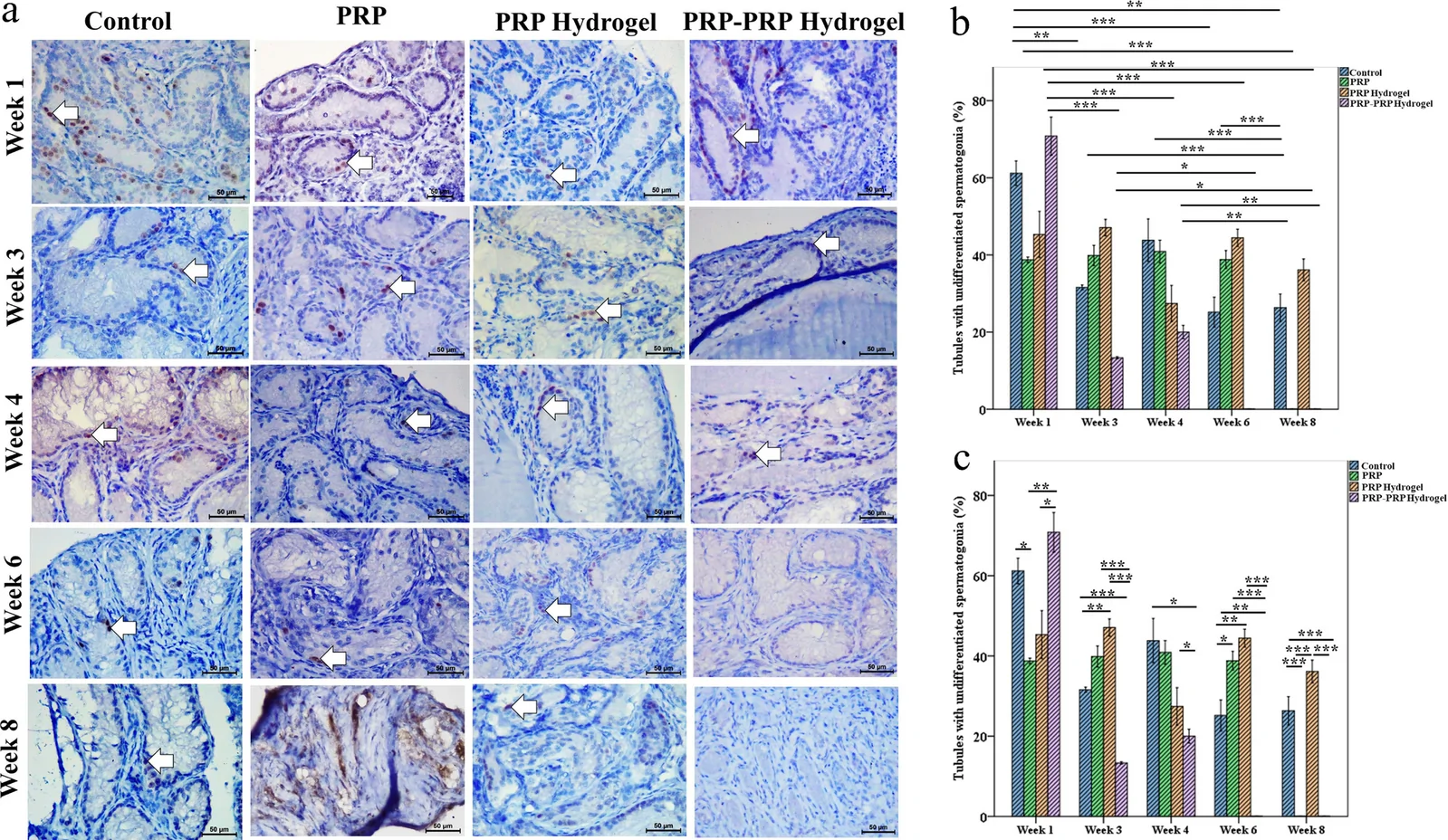
Evaluation of PLZF(+) undifferentiated spermatogonia in rat testicular organotypic cultures. (**a**) Representative images of PLZF immunostaining of cultured testicular tissues. White arrows point to PLZF(+) undifferentiated spermatogonia. (**b**) Changes in the percentage of tubules containing PLZF(+) undifferentiated spermatogonia over time within each group. **c** Comparison of the percentage of PLZF(+) tubules between experimental groups at weeks 1, 3, 4, 6, and 8. Data are presented as mean ± SD (*n* = 6 per group). Statistical significance is indicated as *p < 0.05, **p < 0.01, and ***p < 0.001

The maintenance of PLZF(+) undifferentiated spermatogonia showed distinct patterns across the experimental groups during the 8-week culture period. In the control group, the number of seminiferous tubules containing PLZF(+) cells decreased between weeks 1 and 3 (*p* = 0.002) and remained significantly lower at weeks 6 (*p* < 0.001) and 8 (*p* = 0.001) relative to week 1. In the PRP group, the number of PLZF(+) tubules remained relatively stable until week 6, followed by a marked decline at week 8 (*p* < 0.001). In contrast, the PRP hydrogel–supported culture platform was the only group in which PLZF(+) undifferentiated spermatogonia remained detectable throughout the entire 8-week culture period. Conversely, in the PRP-PRP hydrogel group, the number of PLZF(+) tubules decreased significantly between weeks 1 and 3 (*p* < 0.001) and was maintained through week 4, after which PLZF(+) cells were no longer detected at weeks 6 and 8 (Fig. 3a and b).

In terms of group comparisons, the number of PLZF(+) tubules at week 1 in the PRP-PRP hydrogel group was higher than that observed in the PRP (*p* = 0.004) and PRP hydrogel (*p* = 0.016) groups, while control and PRP-PRP hydrogel groups did not differ significantly. By week 3, the PRP hydrogel group exhibited significantly higher numbers of PLZF(+) tubules than the control (*p* = 0.001) and PRP-PRP hydrogel groups (*p* < 0.001). The lowest values were observed in the PRP-PRP hydrogel group. At week 4, comparable numbers of PLZF(+) tubules were observed in the control, PRP, and PRP hydrogel groups. In contrast, the PRP-PRP hydrogel group exhibited significantly lower values than the control (*p* = 0.018) and PRP groups (*p* = 0.037). At week 6, the PRP (*p* = 0.030) and PRP hydrogel (*p* = 0.004) groups exhibited higher numbers of PLZF(+) tubules compared with the control group, whereas PLZF(+) cells were not detected in the PRP-PRP hydrogel group. At week 8, no PLZF(+) were detected in the PRP and PRP-PRP hydrogel groups, whereas the control and PRP hydrogel groups showed comparable numbers of PLZF(+) tubules (Fig. 3a and c).

### *PRP hydrogel supported the long-term maintenance of SCP3(* +*) spermatocytes*

**Fig. 4:**
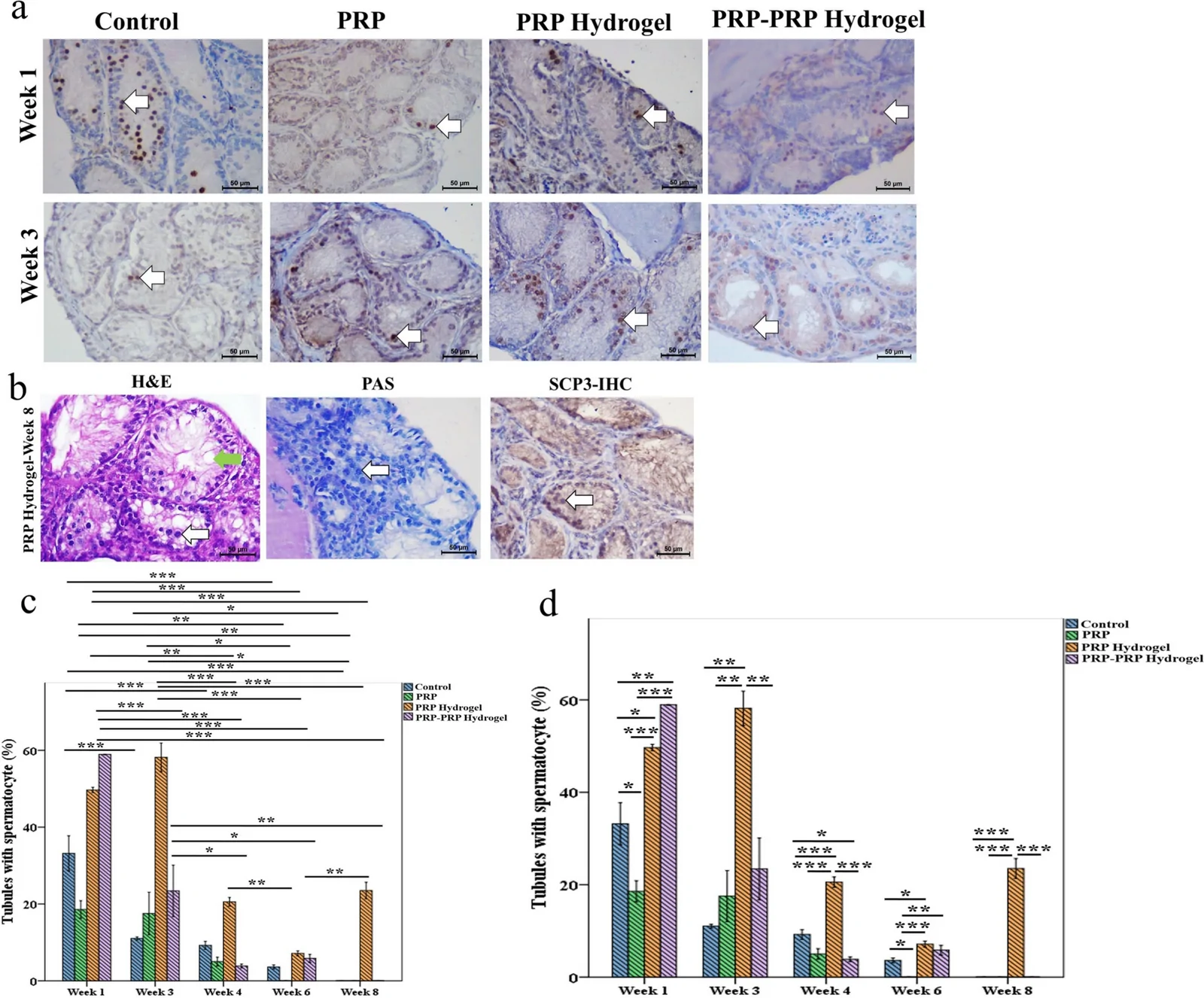
Evaluation of SCP3(+) meiotic germ cells in rat testicular organotypic cultures. (**a**) Representative images of SCP3 immunostaining in cultured testicular tissues. SCP3(+) cells are indicated by white arrows.(**b**) Representative H&E, PAS, and SCP3 immunostaining images of PRP hydrogel–supported cultures at week 8. White arrows indicate SCP3(+) spermatocytes and green arrows indicate lumen-like intratubular structures. (**c**) Time-dependent changes in the percentage of seminiferous tubules containing SCP3(+) cells within each group. (**d**) Comparison of the percentage of SCP3(+) tubules between experimental groups at weeks 1, 3, 4, 6, and 8. Data are presented as mean ± SD (*n* = 6 per group). Statistical significance is indicated as *p < 0.05, **p < 0.01, and ***p < 0.001

The proportion of seminiferous tubules containing SCP3(+) spermatocytes showed varying trends during the culture period. In the control group, the percentage of SCP3(+) tubules declined between weeks 1 and 3 (*p* < 0.001), remained unchanged up to week 6, and was no longer detected at week 8. In the PRP group, SCP3(+) spermatocytes remained stable during the first 4 weeks but were not observed at weeks 6 and 8. In the PRP hydrogel–supported culture platform, the percentage of SCP3(+) tubules remained stable between weeks 1 and 3, with a reduction observed at weeks 4 (*p* < 0.001) and 6 (*p* = 0.009). Notably, an increase was observed at week 8 compared with week 6 (*p* = 0.002), and this group was the only one in which SCP3(+) cells were present at the final time point. In the PRP-PRP hydrogel group, the percentage of SCP3(+) tubules showed a declining trend through week 3 (*p* = 0.001) and week 4 (*p* = 0.011), remaining relatively stable until week 6, with no spermatocytes detected by week 8 (Fig. 4a and b).

Across groups, at week 1, the percentage of SCP3(+) tubules was higher in the PRP hydrogel group than in the control (*p* = 0.012) and PRP (*p* < 0.001) groups, while remaining comparable to the PRP-PRP hydrogel group. At week 3, the PRP hydrogel group exhibited significantly higher percentages of SCP3(+) tubules than the control (*p* = 0.001), PRP (*p* = 0.002), and PRP-PRP hydrogel (*p* = 0.005) groups. A similar pattern was observed at week 4, where the PRP hydrogel group remained significantly higher than all other groups (*p* < 0.001 for all comparisons). At week 6, the PRP hydrogel group showed a significantly higher percentage of SCP3(+) tubules compared with the control (*p* = 0.038) and was comparable to the PRP-PRP hydrogel group, whereas the PRP group showed no detectable SCP3(+) cells. By week 8, SCP3(+) spermatocytes were detected exclusively in the PRP hydrogel group, whereas no SCP3(+) cells were observed in the other experimental groups (Fig. 4a and c).

### No detectable ACR expression in rat testicular organotypic cultures

**Fig. 5:**
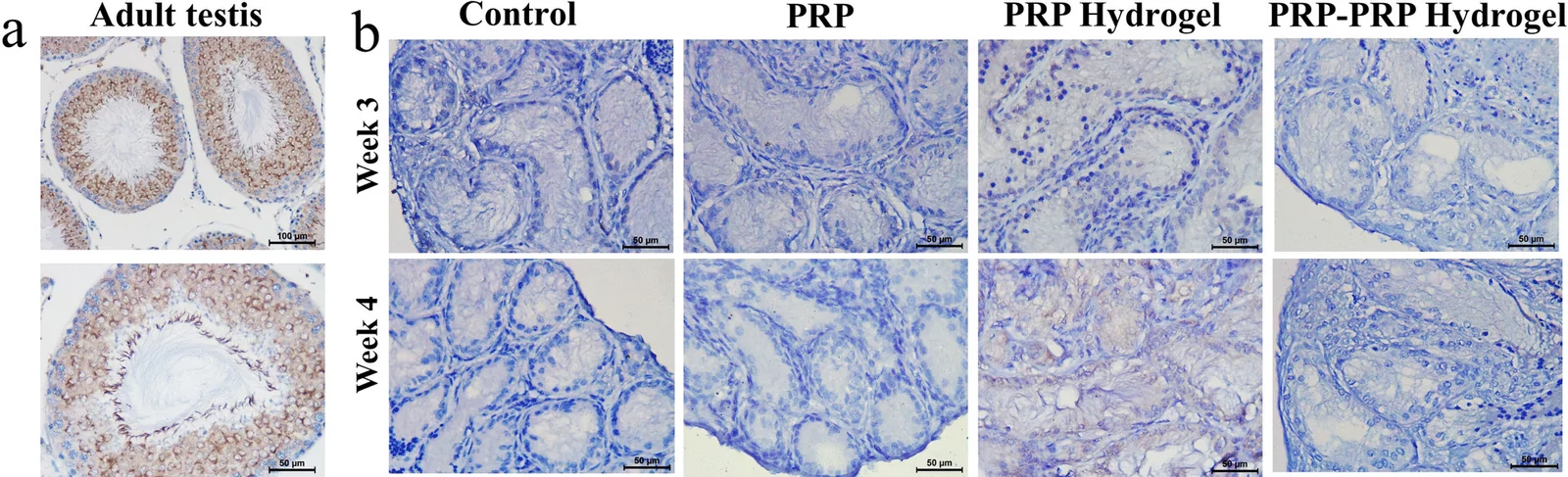
Evaluation of ACR expression in rat testicular organotypic cultures. (**a**) Adult rat testicular tissue was used as a positive control for acrosin immunostaining. (**b**) Representative images of ACR immunostaining in cultured testicular tissues. No ACR(+) cells were detected under the examined conditions. Scale bar = 50 µm

According to immunostaining results, ACR(+) post-meiotic germ cells were not detected in any experimental group throughout the culture period (Fig. 5).

## Discussion

The present study investigated the potential of a PRP-based organotypic culture system to support spermatogenic progression and germ cell maintenance during long-term culture. While organotypic culture systems have been widely utilized to study testicular function, replicating the complex in vivo microenvironment required for sustained germ cell survival and differentiation remains a major challenge. Although complete in vitro spermatogenesis has been successfully achieved in mouse models, maintaining germ cell survival and differentiation in other species has proven considerably more difficult [7]. In this context, our findings suggest that utilizing PRP as a three-dimensional hydrogel scaffold may provide a more supportive microenvironment for long-term organotypic culture of testicular tissue. Notably, the persistence of PLZF(+) spermatogonia and SCP3(+) spermatocytes observed in PRP hydrogel–supported cultures indicates that this scaffold may contribute to maintaining germ cell populations and supporting meiotic activity during extended culture periods. Furthermore, the better preservation of seminiferous tubule architecture, gradual enlargement of tubular area, and higher frequency of lumen-like intratubular structures observed in PRP hydrogel–supported cultures suggest that this scaffold may provide structural and biochemical cues that support germ cell maintenance within the seminiferous epithelium. Rather than merely preserving the initial cell population, this three-dimensional microenvironment appears to provide a more favorable niche for sustaining germ cell populations during long-term culture. To our knowledge, this study represents the first evaluation of a PRP hydrogel scaffold in a rat-based testicular organotypic culture model.

Platelet-rich plasma is known to contain a wide range of bioactive molecules, including growth factors and cytokines that regulate cellular proliferation, survival, and tissue regeneration [11]. Owing to these biological properties, the potential of PRP to support germ cell populations has also been explored in studies focusing on SSC cultures in vitro. Khadivi et al. demonstrated that supplementation of culture medium with 5% PRP promoted the proliferation of human SSCs in a two-dimensional culture system. In the same study, PRP was also used as a three-dimensional hydrogel scaffold, which supported SSC proliferation and showed higher expression of SSC markers GFRA1 and c-KIT [22]. These findings suggest that PRP-based scaffolds may provide a supportive microenvironment for maintaining SSC characteristics. Similarly, Salem et al. reported that culturing SSCs on a decellularized testicular matrix supplemented with PRP enhanced cell viability while maintaining the expression levels of PLZF and SCP3, and increasing the expression of the differentiation marker PRM2 [23]. In another study comparing PRP with conventional serum-based culture conditions, the expression levels of SSC markers such as PLZF, OCT4, and c-KIT were reported to be comparable between cultures supplemented with 10% PRP and those containing 10% FBS.

While these previous studies primarily focused on isolated human SSC cultures, the present study employed a rat testicular organotypic culture system that preserves the native seminiferous tubule architecture and cell–cell interactions within the testicular niche. Despite these methodological differences, the findings collectively support the concept that PRP-based microenvironments may contribute to germ cell survival. In the present study, however, the most consistent effects were observed when PRP was used as a hydrogel scaffold rather than solely as a culture medium supplement. PRP hydrogel–supported cultures maintained PLZF(+) undifferentiated spermatogonia and sustained SCP3(+) spermatocytes during long-term culture, with both cell populations remaining detectable at week 8. Although the combined condition of PRP supplementation with PRP hydrogel showed partially comparable results at certain time points, the PRP hydrogel scaffold alone provided the most stable environment for maintaining germ cell populations over the entire culture period. These findings suggest that the structural properties of the PRP hydrogel scaffold may play a critical role in creating a supportive niche for germ cell maintenance in organotypic culture systems.

The therapeutic potential of PRP has been demonstrated in several in vivo models of testicular injury, where PRP administration has been reported to support tissue recovery and germ cell survival following chemical or physical damage [13–21]. However, despite these promising in vivo findings, the application of platelet-derived products in in vitro testicular organotypic culture systems remains limited. In most available studies, these products have been used primarily as culture medium supplements rather than as structural scaffolds. Moreover, the majority of these investigations have been conducted using mouse models, leaving limited information on the applicability of these approaches to more challenging species [24, 25]. In one such study, immature testicular tissues obtained from mice were cultured for the first in media supplemented with different concentrations of platelet-rich growth factors (PRGF) (5%, 10%, 15%, and 20%), and the authors reported that cultures maintained with 5% PRGF were able to support spermatogenesis up to the flagellated sperm stage, whereas such advanced differentiation was not observed in the control cultures [24].

However, the experimental design of that study differs from the present work in several important aspects. First, regarding the source of platelet-derived products, the previous study utilized human-derived PRGF for mouse testicular tissue, whereas the present study employed a species-matched approach by using rat-derived PRP for rat testicular cultures. The use of species-specific platelet-derived products may provide a more physiologically relevant microenvironment for germ cell maintenance. Second, while the previous work evaluated PRGF primarily as a soluble medium supplement, the present study also investigated PRP as a three-dimensional hydrogel scaffold during long-term culture. Furthermore, species-related differences should also be considered. Complete in vitro spermatogenesis has been achieved more successfully in mice than in other species. These inherent interspecies differences may partly explain the limited progression toward post-meiotic stages observed in rat-based systems compared with the results obtained in mice. Nevertheless, our findings demonstrate that the PRP hydrogel scaffold effectively maintained PLZF(+) spermatogonia and SCP3(+) spermatocytes for extended periods. Taken together, these methodological differences highlight the potential of PRP-based three-dimensional microenvironments as a promising strategy for improving long-term germ cell persistence in rat testicular organotypic culture models.

The same research group also evaluated the combined use of PRGF and KSR in mouse testicular organotypic cultures. In that study, testicular tissues derived from mice at postnatal day 5 were cultured for 42 days in medium supplemented with 5% KSR and 5% PRGF, while the control group was maintained in medium containing 10% KSR. Although spermatogenesis progressed to post-meiotic stages in the control cultures, the combined condition resulted in a general failure of the culture system characterized by a significant loss of germ cells including the PLZF(+) and SCP3(+) populations and a complete absence of post-meiotic markers [25]. These observations suggest that differences in culture conditions and PRGF application strategies may influence the effects of platelet-derived products on germ cell maintenance. While PRGF supplementation alone was previously reported to support spermatogenic progression in mouse cultures, the combined PRGF-KSR condition resulted in impaired germ cell survival and loss of post-meiotic differentiation. In contrast, our findings suggest that providing PRP-derived factors within a structural hydrogel scaffold may offer a more stable and supportive microenvironment capable of sustaining germ cell populations throughout the 8-week period. In addition, the enriched culture medium composition used to support long-term rat organotypic culture may also have influenced tissue responses and potentially masked certain PRP-related effects. Future studies using simplified or stepwise-defined culture conditions may help further clarify the specific contribution of PRP-derived factors.

In conclusion, the present study demonstrates that PRP hydrogel can function as a supportive three-dimensional scaffold in rat testicular organotypic culture systems. Species-matched PRP hydrogel scaffolds may represent a supportive platform for maintaining germ cell populations during long-term culture. These findings suggest that PRP-based three-dimensional microenvironments may represent a promising strategy for improving in vitro testicular tissue culture platforms and may provide a potential experimental framework for future fertility preservation approaches.

### Limitations

Despite the encouraging findings of the present study, certain limitations should be taken into account. First, although PRP hydrogel–supported cultures were able to maintain PLZF(+) spermatogonia and SCP3(+) spermatocytes during long-term culture, complete spermatogenic progression to post-meiotic stages was not achieved in the rat organotypic culture system. This limitation is consistent with previous studies indicating that in vitro spermatogenesis is considerably more challenging in rats than in mice. Second, the present study focused primarily on histological and immunohistochemical evaluation of germ cell populations, and additional molecular analyses may provide further insight into the mechanisms by which PRP-derived factors influence germ cell maintenance. In addition, PRP-derived growth factors and bioactive molecules were not directly quantified in the present study, and further characterization of their release dynamics within the culture system may provide mechanistic insight into the observed tissue responses. Finally, although PRP hydrogel provided a supportive three-dimensional microenvironment for testicular tissue fragments, further optimization of culture conditions, including medium composition and culture duration, as well as inclusion of additional non-PRP hydrogel control systems, may help better distinguish the relative contributions of hydrogel architecture and PRP-derived bioactive factors while supporting full spermatogenic differentiation in rat-based organotypic culture systems.

## Data Availability

The dataset generated and analyzed during the current study are available from the corresponding author upon reasonable request.

## References

[1] Salem M, Khadivi F, Javanbakht P, Mojaverrostami S, Abbasi M, Feizollahi N, et al. Advances of three-dimensional (3D) culture systems for in vitro spermatogenesis. Stem Cell Res Ther. 2023;14(1):262. 37735437 10.1186/s13287-023-03466-6 PMC10512562

[2] Mohammadi A, Bashiri Z, Rafiei S, Asgari H, Shabani R, Hosseini S, et al. Testicular niche repair after gonadotoxic treatments: current knowledge and future directions. Biol Cell. 2024;116(4):e2300123. 38470182 10.1111/boc.202300123

[3] Pasten Gonzalez A, Salvador Alarcon C, Mora J, Martin Gimenez MP, Carrasco Torrents R, Krauel L. Current status of fertility preservation in pediatric oncology patients. Children. 2024. 10.3390/children11050537. 38790532 PMC11120648

[4] Reda A, Hou M, Winton TR, Chapin RE, Soder O, Stukenborg JB. In vitro differentiation of rat spermatogonia into round spermatids in tissue culture. Mol Hum Reprod. 2016;22(9):601–12. 27430551 10.1093/molehr/gaw047 PMC5013872

[5] Saulnier J, Soirey M, Kebir N, Delessard M, Rives-Feraille A, Moutard L, et al. Complete meiosis in rat prepubertal testicular tissue under in vitro sequential culture conditions. Andrology. 2023;11(1):167–76. 36303516 10.1111/andr.13325 PMC10099474

[6] Saulnier J, Oblette A, Delessard M, Dumont L, Rives A, Rives N, et al. Improving freezing protocols and organotypic culture: a histological study on rat prepubertal testicular tissue. Ann Biomed Eng. 2021;49(1):203–18. 32440757 10.1007/s10439-020-02535-8

[7] Matsumura T, Sato T, Abe T, Sanjo H, Katagiri K, Kimura H, et al. Rat in vitro spermatogenesis promoted by chemical supplementations and oxygen-tension control. Sci Rep. 2021;11(1):3458. 33568686 10.1038/s41598-021-82792-2 PMC7875995

[8] Matsumura T, Katagiri K, Yao T, Ishikawa-Yamauchi Y, Nagata S, Hashimoto K, et al. Generation of rat offspring using spermatids produced through in vitro spermatogenesis. Sci Rep. 2023;13(1):12105. 37495678 10.1038/s41598-023-39304-1 PMC10372019

[9] Nakamura N, Sloper DT. Comparison of germ cell differentiation of rat testis fragments cultured in knockout serum replacement versus Albumax I. Birth Defects Res. 2021;113(4):359–70. 33348473 10.1002/bdr2.1859

[10] Liu F, Cai C, Wu X, Cheng Y, Lin T, Wei G, et al. Effect of KnockOut serum replacement on germ cell development of immature testis tissue culture. Theriogenology. 2016;85(2):193–9. 26474686 10.1016/j.theriogenology.2015.09.012

[11] Rath M, Spinnen J, Kuhrt LD, Priglinger E, Seika P, Runge D, et al. Platelet-rich plasma - a comprehensive review of isolation, activation, and application. Acta Biomater. 2025;204:52–75. 40712724 10.1016/j.actbio.2025.07.050

[12] Moradian SA, Amirkhani Z, Movahedin M, Gharib SN, Varghaiyan Y, Niknafs B, et al. Platelet-rich plasma (PRP) and the future of male fertility: a path forward for personalized and regenerative therapies. Stem Cell Res Ther. 2025;16(1):486. 40898310 10.1186/s13287-025-04602-0 PMC12403957

[13] Dehghani F, Sotoude N, Bordbar H, Panjeshahin MR, Karbalay-Doust S. The use of platelet-rich plasma (PRP) to improve structural impairment of rat testis induced by busulfan. Platelets. 2019;30(4):513–20. 29883240 10.1080/09537104.2018.1478400

[14] Samy A, El-Adl M, Rezk S, Marghani B, Eldomany W, Eldesoky A, et al. The potential protective and therapeutic effects of platelet-rich plasma on ischemia/reperfusion injury following experimental torsion/detorsion of testis in the Albino rat model. Life Sci. 2020;256:117982. 32562693 10.1016/j.lfs.2020.117982

[15] Sayed WM, Elzainy A. Impact of platelet-rich plasma versus selenium in ameliorating induced toxicity in rat testis: histological, immunohistochemical, and molecular study. Cell Tissue Res. 2021;385(1):223–38. 33791879 10.1007/s00441-021-03439-2

[16] Kutluhan MA, Ozsoy E, Sahin A, Urkmez A, Topaktas R, Toprak T, et al. Effects of platelet-rich plasma on spermatogenesis and hormone production in an experimental testicular torsion model. Andrology. 2021;9(1):407–13. 32866352 10.1111/andr.12895

[17] Demyashkin GA, Borovaya TG, Andreeva YY, Nedorubov AA, Stepanova YY, Vadyukhin MA, et al. An experimental approach to comprehend the influence of platelet rich growth factors on spermatogenesis. Int J Radiat Biol. 2022;98(8):1330–43. 35259048 10.1080/09553002.2022.2047820

[18] Demyashkin GA, Vadyukhin MA, Shekin VI. The influence of platelet-derived growth factors on the proliferation of germinal epithelium after local irradiation with electrons. J Reprod Infertil. 2023;24(2):94–100. 37547573 10.18502/jri.v24i2.12494 PMC10402459

[19] Kim S, Kim JM, Jeon EJ, Kim JW, Choi ME, Park JM, et al. Supernatant of activated platelet-rich plasma rejuvenated aging-induced hyposalivation in mouse. Sci Rep. 2023;13(1):21242. 38040732 10.1038/s41598-023-46878-3 PMC10692196

[20] Saba AI, Elbakary RH, Afifi OK, Sharaf Eldin HEM. Effects of platelet-rich plasma on the oxymetholone-induced testicular toxicity. Diseases (Basel). 2023. 10.3390/diseases11020084. PMC10297451 37366872

[21] Abdel Ghaffar DM, Eldken ZH, Sultan MS, Khalil RM, Sakr NH, Eissa H, et al. Insights on protective effect of platelet rich plasma and tadalafil on testicular ischemia/reperfusion injury in rats exposed to testicular torsion/detorsion. Cell Physiol Biochem. 2024;58(1):14–32. 38232236 10.33594/000000680

[22] Khadivi F, Koruji M, Akbari M, Jabari A, Talebi A, Ashouri Movassagh S, et al. Application of platelet-rich plasma (PRP) improves self-renewal of human spermatogonial stem cells in two-dimensional and three-dimensional culture systems. Acta Histochem. 2020;122(8):151627. 33002788 10.1016/j.acthis.2020.151627

[23] Salem M, Feizollahi N, Jabari A, Golmohammadi MG, Shirinsokhan A, Ghanami Gashti N, et al. Differentiation of human spermatogonial stem cells using a human decellularized testicular scaffold supplemented by platelet-rich plasma. Artif Organs. 2023;47(5):840–53. 36721957 10.1111/aor.14505

[24] Moradian SA, Movahedin M. In vitro sperm generation from immature mouse testicular tissue using plasma rich in growth factors. Stem Cell Res Ther. 2025;16(1):17. 39849580 10.1186/s13287-025-04136-5 PMC11755862

[25] Moradian SA, Khaledi S, Amirkhani Z, Movahedin M. Combination of knockout serum replacement and plasma rich in growth factors does not support in vitro spermatogenesis in mice. Sci Rep. 2025;15(1):31506. 40858886 10.1038/s41598-025-16502-7 PMC12381235

